# Creative Motor Actions As Emerging from Movement Variability

**DOI:** 10.3389/fpsyg.2017.01903

**Published:** 2017-10-31

**Authors:** Dominic Orth, John van der Kamp, Daniel Memmert, Geert J. P. Savelsbergh

**Affiliations:** ^1^Amsterdam Movement Sciences, Faculty of Behavioural and Movement Sciences, Vrije Universiteit Amsterdam, Amsterdam, Netherlands; ^2^Institute for Brain and Behavior, Amsterdam, Netherlands; ^3^Institute of Training and Computer Science in Sport, German Sport University Cologne, Cologne, Germany

**Keywords:** motor creativity, motor skill, constraints, transfer, learning, coordination, degeneracy, exploration

## Abstract

In cognitive science, creative ideas are defined as original and feasible solutions in response to problems. A common proposal is that creative ideas are generated across dedicated cognitive pathways. Only after creative ideas have emerged, they can be enacted to solve the problem. We present an alternative viewpoint, based upon the dynamic systems approach to perception and action, that creative solutions emerge *in* the act rather than *before*. Creative actions, thus, are as much a product of individual constraints as they are of the task and environment constraints. Accordingly, we understand creative motor actions as functional movement patterns that are new to the individual and/or group and adapted to satisfy the constraints on the motor problem at hand. We argue that creative motor actions are promoted by practice interventions that promote exploration by manipulating constraints. Exploration enhances variability of functional movement patterns in terms of either coordination or control solutions. At both levels, creative motor actions can emerge from finding new and degenerate adaptive motor solutions. Generally speaking, we anticipate that in most cases, when exposed to variation in constraints, people are not looking for creative motor actions, but discover them while doing an effort to satisfy constraints. For future research, this paper achieves two important aspects: it delineates how adaptive (movement) variability is at the heart of (motor) creativity, and it sets out methodologies toward stimulating adaptive variability.

## Introduction: Traditional Accounts of Creativity

In the cognitive sciences, creativity is typically conceived as the expression of original, yet feasible ideas, insights and solutions for problems (Guilford, 1956). It is argued to play a significant role in advancing and transforming any given field of human endeavor (Runco, 2004; Memmert, 2015; Asma, 2017). Accordingly, scientists have long been interested in understanding the source of creativity.

A major challenge, however, is the difficulty posed in observing creativity (Runco, 2004). The creative ideas that advance or transform human endeavor are few and far between. They are unlikely to occur within the walls of a science laboratory (Sternberg et al., 2001). In overcoming this, scientists developed empirical approaches that require an individual participant to generate as many ideas as possible to solve a certain problem, the assumption being that creative ideas arise – in some manner – from the variation in ideas. The larger the variability, the more likely that a creative idea is among it. Accordingly, the amount (termed fluency) and originality of ideas are then taken as makeshift measures for creativity (De Dreu et al., 2012). In many cases, these measures are compared across individuals and correlated to individual characteristics (e.g., working memory and attention) (Simonton, 2003; De Dreu et al., 2012; Lin and Lien, 2013). The associations have subsequently (and perhaps prematurely) been interpreted as evidence for the existence of cognitive pathways or systems that cause creative ideas (Nijstad et al., 2010). Indeed, this proposition that creativity is caused by the operation of specialized cognitive systems has underpinned the search for neural systems that enhance or inhibit (Dietrich and Kanso, 2010; Kounios and Beeman, 2014).

We challenge the underlying assumption of this (traditional) account of creativity: the notion that individuals first generate an idea in their mind, which is then enacted in behavior (this notion is also referred to as hylomorphism, Ingold, 2013). The main problem with this notion is that it can falsely lead to the inference that creativity *is* ideation and that the action is simply an expression of the creativity process, rather than it being *part or constitutive* of creativity. Indeed, recent evidence shows that ideation or the generation of creative ideas depend on contextual characteristics (Lin and Lien, 2013). For example, tasks with many solutions (i.e., divergent thinking tasks) normally encourage flexible-like outcomes (where more ideas across cognitive categories occur), whereas tasks with few or a single solution (i.e., convergent tasks) encourage persistent-like outcomes (where more ideas within cognitive categories occur). This suggests that creativity is also underpinned by how characteristics of the task interact with the individual to constrain possible solutions (Simonton, 2003).

Within dynamical systems (Kelso, 1995) and ecological approaches (Gibson, 1979), actions are considered as emergent in the temporary couplings formed among the individual and the environment (Newell, 1986; Thelen, 1995; Davids et al., 2008). Importantly, these couplings are not uniquely determined by the individual’s characteristics, but in unity with environmental and task constraints. These constraints define the space within which the movement system can act, placing boundaries on the movement solutions available (Sporns and Edelman, 1993; Newell et al., 2003). From this perspective, creative motor actions are as much a function of the individual, as the task and environment (Hristovski et al., 2011). They can arise in the temporal coupling between the organism and environment, while the action unfolds. Thus, rather than referring to ideas that are uniquely generated by a (creative) cognitive system, we use the term creative as a descriptive for unfolding actions that are original (relative to the individual or group) and functional (i.e., they support task success) (see also, Hristovski et al., 2011).

In sum, we challenge a number of core theoretical and methodological assumptions in current creativity research. Chief among these is the -in our opinion- incorrect assumption that creative ideas are independent of behavior such that the creative mental idea is seen to cause actions that are creative. A major problem with this assumption is that it has resulted in a narrow set of experimental tasks (often jotting down ideas on paper), which are largely restricted to one-off observations that insufficiently account for individual experiential histories (including prior deliberate practice activities). In this paper, we use dynamics systems and ecological approaches to propose an alternative framework for theorizing and assessing how constraints on practice influence the emergence of creative actions.

## A ‘New’ Approach to Creativity: on Movement Variability and Adaptability

The importance placed on creativity in motor actions is in practical terms no different to any other field of human endeavor. For example, the ‘Fosbury flop,’ which is now the dominant technique used in competitive high jump (and discussed later in this section) is an eminent example of how creativity in motor actions can advance performance and reshape the way an activity is practiced (Hristovski et al., 2011). Often referred to as motor creativity (Wyrick, 1968; Memmert, 2007), the capability of individuals to show original and functional motor actions is considered as an important aspect of skill and adaptability (Hristovski et al., 2011; Davids et al., 2015) or, as referred to by Bernstein, dexterity. According to Bernstein (1996), dexterity denotes “finding a motor solution for any situation and in any condition” (Bernstein, 1996, p. 21)… where… “demand for dexterity is not in the movements themselves but in [adapting to] the surrounding conditions” (Bernstein, 1996, p. 23). These ideas have led toward and understanding of how movement variability is at the heart of this adaptability to any dynamic context (Riley and Turvey, 2002; Button et al., 2003; Liu et al., 2006). Accordingly, within the overall distribution of adaptive motor solutions, creative motor actions refer to solutions that are (statistically) rare and thus original (Simonton, 2003; Memmert and Perl, 2009). We argue, that motor creativity reflects an individual’s adaptability, but is exceptional in its level of originality relative to other adaptive solutions (i.e., within and/or between individuals). Motor creativity can be defined as new ways of acting adaptive, or acting adaptive in new situations (Hristovski et al., 2011). In both instances, this implies that creative actions are functional.

On the behavioral level, there is generally an insight into how functional the action is. For example, Newell’s proposal on the emergence of skill (Newell, 1986) suggests that movement variability is functional if it ensures that action goals can be met by the individual, such that performance is maintained as constraints are changed (see also, Davids et al., 2003). Functionality, thus, can be understood in terms of task success – how hard someone hits, how accurate they aim, how efficiently they move, etc. However, there is no absolute criterion for originality. If creative motor actions are granted by functional or adaptive movement variability, it also needs to be reconciled with Bernstein’s insight, that, depending on the analysis level, each movement pattern can be considered unique: ‘repetition without repetition’ (Bernstein, 1967; Riley and Turvey, 2002). Pragmatically, originality can be taken to mean ‘more or less’ different from previous movement patterns where the frame of reference can be at the within individual, inter-individual or social levels. At the individual level, behaviors can be considered unique relatively to the individual’s previous movement patterns (Hristovski et al., 2011). At the inter-individual and social levels, originality is judged in terms of differences between experimental groups (Moraru et al., 2016) or against the extant social-cultural background (Boden, 1998; Immonen et al., 2017). In this paper we focus on originality at the individual and inter-individual levels, since they are currently more feasible in the experimental context (Boden, 1994; Beghetto and Kaufman, 2007) and we may assume that inter-individual creativity might emerge from interactions observed at the individual level (Gillespie et al., 2014). In the future, innovations in understanding how social interactions shape creativity, such as by adapting big data approaches, may open up a way to concurrently address these levels of analysis (Gillespie et al., 2014).

The problem of determining the originality of actions relative to the individual is addressed in motor learning studies that examine individuals or groups acquiring new motor skills. Particularly in beginners, but potentially also in experts, original and functional actions can emerge in practice due to an effort to improve performance (Vereijken et al., 1992; Delignières et al., 1998; Liu et al., 2006). According to Newell (1996, p. 398), “a skilled performer changes the solution to the movement coordination and control problem according to the various changing demands of the organism-environment interaction and to the pursuit of the task goal.” Hence, the source of variability -and hence originality- can refer to coordination solutions or distinctive patterns or classes of movement such as walking or running as ways of locomotion. Yet, variability can also emerge from variability in control solutions, which refers to movements made that regulate the stability of a given coordinative solution (Newell, 1986, 1996).

Traditionally, the terms coordination and control are also used to describe a general progression of how the individual changes behavior during learning under a set of constraints. Here, the learner initially seeks out a stable coordination solution (the coordination stage), which is then refined and improved through practice (the control stage). Coordination is defined as the function that constrains the available movement system degrees of freedom into a functional movement pattern. Control refers to the parameterizing of the topological relations of the coordination pattern formed between parts of the human movement system (Newell, 1985). It is important to recognize that these two concepts are inextricable linked (or embedded) in that coordination occurs with control and vice versa (Newell, 1996). Furthermore, we would also emphasize that constraints are never truly fixed from one trial to the next (Rosalie and Müller, 2012) and that learning is not accurately summarized as progressing in a linear stage-like fashion (Chow et al., 2011).

**FIGURE 1:**
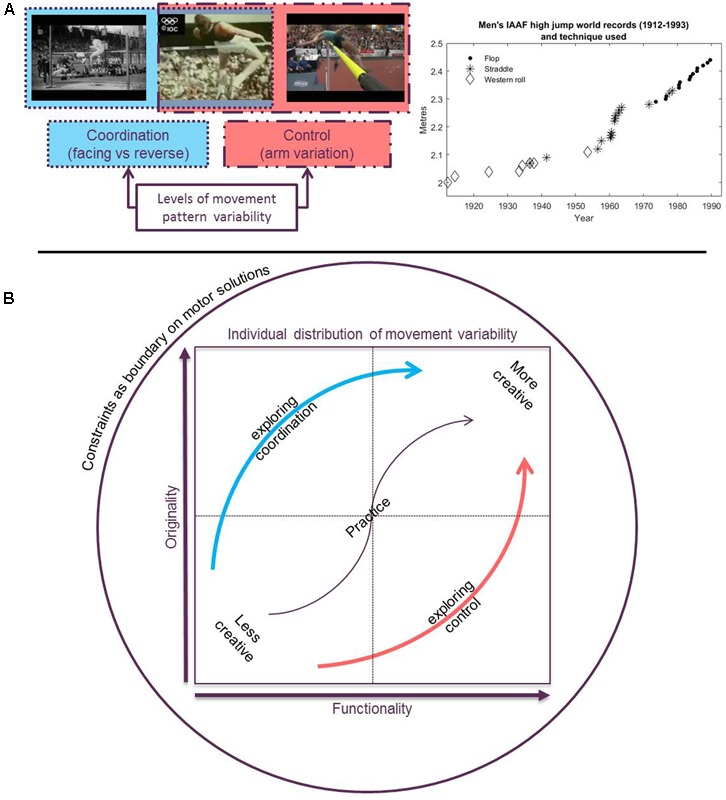
Creative movement behavior granted by variability in coordination and control under constraint. **(A)** Shows the relationship between creative motor actions and movement variability at levels of coordination and control during practice. In the high jump, changing the technique from scissor jump to the flop involves providing a new coordination solution (the way angles/joints are constrained are clearly different, as can be seen as well in how the body is oriented relative to the bar). In improving the flop the arm does not fundamentally change these linkages, rather is a remaining degree of freedom that is parameterized to optimize performance further. Hence, relative to the original flop this is a new control solution. The boxes overlap because coordination and control solutions are embedded, they are always interdependent. The graph on the right shows the relationship between jumping techniques and performance over time. **(B)** Shows how exploration during practice can give rise to new and functional behavior (i.e., creative motor actions) at levels of either coordination or control. Either level can give rise to creative motor actions and may be emphasized at different points during practice (i.e., as depicted by the central arrow). Note that this diagram refers to the emergence of creative actions at the individual level, but can also come to represent the actions new to society (e.g., when Fosbury created his flop). IAAF, International Association of Athletics Federations. High jump images source: https://www.youtube.com/watch?v=RUfCCo_x1oI. High jump record data source: https://en.wikipedia.org/wiki/Men%27s_high_jump_world_record_progression. High jump technique data source: https://www.thoughtco.com/mens-high-jump-world-records-3258798.

The discovery by the learner of new coordination and control solutions during practice has been linked to improvements in performance (Liu et al., 2006). As an eminent example, **Figure 1A**, shows how different coordination and control solutions have led to improved performance in high jump over the years. In one instance, Dick Fosbury won gold in the 1968 Mexico Olympics high jump event by clearing the bar head first and with his back facing it. This was a new coordination solution, a qualitatively new movement pattern (most likely on the social level) compared to the other and previous competitors in the discipline who typically cleared the bar either side on (the so called ‘western roll’) or face down (the ‘straddle’). Indicative of its significant social impact, the ‘Fosbury flop’ remains to be the dominant technique used at competitive level today. Such creative motor actions emerge not by optimizing a well-established technique, but through the adaptation of a qualitatively new movement pattern (relative to the ‘western roll’ and ‘straddle’) to changes in constraints (Hristovski et al., 2011). In this case, the ‘flop’ emerged alongside the implementation of high density foam safety mats, allowing for a soft landing on the shoulders (Farrow and Kemp, 2006). Also notable, however, is the substantial inter-individual variability related to the same coordination solution since 1968. One example of different control solutions relative to the original solution by Fosbury is given **Figure 1A**, which illustrates the use of arm movements that help to optimize, propelling the body’s center of mass higher (Lees et al., 2000).

Henceforth, a full appreciation of motor creativity includes addressing these respective transformative (changes in coordination) and optimization (changes in control) processes (Boden, 1998; Hristovski et al., 2011). In **Figure 1B**, the traditional portrayal of creative solutions is integrated with the constraints based motor learning framework by indicating the two key axes along which creative outcomes are typically judged – novelty and functionality. New and functional solutions are discovered in terms of new coordination patterns and/or the way these are controlled. The new solutions are discovered and shaped during practice and exploration is necessary (Newell et al., 1989). Constraints on practice may guide the individual toward a greater number of qualitatively different solutions (the coordination pathway of the blue arrow in **Figure 1B**) or promote the in depth exploration of a single or fewer solutions (the control pathway of the red arrow in **Figure 1B**).

## Creative Coordination and Control Solutions Emerge Through Exploration Under Constraints

To briefly summarize, we have proposed that creative motor actions are granted by functional and adaptive movement variability both at the level of coordination and control. The search for adaptive solutions is the impetus for finding original and functional motor solutions (i.e., corresponding to current definitions of creative outcomes). This search or exploration is continuous emerging over different timescales; it occurs whenever an action is produced, but perhaps more deliberately during practice (having implications for a diverse range settings such as rehabilitation, health and fitness, sport and physical activity), or across and in-between practice sessions (Newell et al., 2001; Rosengren et al., 2003). We propose therefore that motor learning is a paradigm par excellence for observing and examining how different constraints coalesce to influence the emergence of creative motor actions.

The extensive practice that leads to dexterity brings enhanced adaptability or functional movement variability, a key characteristic of expert behavior (Newell, 1996; Hristovski et al., 2011). Movement variability at the coordination and/or control levels is typically increased by changes in constraints during practice, either occurring naturally or through manipulation (Stergiou et al., 2006; Schöllhorn et al., 2009; Davids et al., 2015). Potentially, such increases in movement variability also increase the likelihood of creative (i.e., statistically rare and adaptive) coordination and/or control solutions (Simonton, 2003). The coach (or teacher, therapist or experiment) can thus encourage more or less variability (and therefore motor creativity) by manipulating the constraints during practice (Ranganathan and Newell, 2013; Buszard et al., 2016; Orth et al., 2016).

Inducing changes in constraints that have a significant influence over the stability or functionality of the coordination solution requires transitions across different patterns of coordination for maintaining success (Hristovski et al., 2011; Kelso, 2012). In basketball, for example, positioning an individual further from the basket, requires him/her to reorganize the nature of the action used, for instance, by using two hands instead of one, to put more strength into the throw (Rein et al., 2010; Ranganathan and Newell, 2013). In this case the practitioner facilitates the exploration of a greater number of coordination solutions during practice, perhaps with an initial but temporary trade-off of functionality. The additional benefit of this approach is that, after practice, beyond having found new functional solutions, the individual may find it easier to reorganize their movement system under new constraints (Kostrubiec et al., 2012; Seifert et al., 2015; Orth et al., 2017). They may transfer their skill and learning better under constraints that require flexibility across coordinative solutions (for definitions see, Carroll et al., 2001).

On the other hand, the exploration of an existing pattern of coordination can be encouraged by constraints that promote the practice of a single or a small number of solutions, referred to by Ranganathan and Newell (2013) as exploration of execution redundancy. In basketball by modifying the lateral position to the basket such that the absolute distance to the basket does not change, the individual is encouraged to use the same technique, but must make local and unplanned adaptations to variations in body positioning relative to the basket, which are independent of distance. Practicing a range of control solutions is generally associated with a more progressive or linear degree of performance improvement. In this case, the benefit to the learner of exploring a single or few coordinative patterns is that functionality may improve faster, which is perhaps traded against a reduction in novelty at the coordination level. So long as constraints in new contexts support the practiced control solutions or if these solutions can be used to learn in new contexts, the individual can transfer their skill and learning effectively to new contexts (Seifert et al., 2016b).

By developing through practice, multiple stable coordination and control solutions, system degeneracy is increased (Kelso, 2012; Seifert et al., 2016a). At the level of motor action, increased system degeneracy means that the individual has developed multiple (and dissimilar) motor solutions for achieving the same outcome or function (Edelman and Gally, 2001; Kelso, 2012; Mason et al., 2015). The manipulation of task and environmental constraints relative to the individual’s current capabilities can thus serve to increase and shape system degeneracy (Rosengren et al., 2003). Practice under changing constraints influences the coordination and control solutions that are learnt and their transferability (Harrison and Stergiou, 2015; Pesce et al., 2016). More importantly, the changing of constraints functions to structure the search or exploration during learning, and it is especially during this exploration -we propose- that new and original coordination and control solutions are found.

Through a careful design of the practice and performance contexts, motor problems invites an individual to explore a range of possible coordination solutions, which is maybe analog to exploring flexible solutions across cognitive categories in divergent thinking tasks (for definitions see, Dietrich and Kanso, 2010; Moraru et al., 2016). Alternatively, encouraging an in-depth exploration of a single or narrow sub-set of techniques or control solutions would be similar to how convergent thinking tasks (for definitions see, Kounios and Beeman, 2014) induce persistent solutions within the same or a reduced subset of cognitive categories (Nijstad et al., 2010). Yet, rather than invoking different (cognitive) pathways to explain the emergence of new coordination and control solutions, these solutions are explained as a temporary (re)organization of the movement system in adaptation to constraints (Newell, 1991, 1996). We now draw on these ideas for proposing a set of theoretical assumptions and specific experimental strategies to understand and stimulate the emergence of creative motor actions.

## Proposed Operational Framework and Future Challenges

Our proposal for understanding motor creativity is underpinned by several assumptions:

(a) Appropriate complexity or difficulty needs to be embodied in the constraints upon action for creative motor action to emerge.
(b) Exploration emerges as an active process to satisfy (changes in and between) a coalition of personal, task and environmental constraints and brings about an increase in movement variability (i.e., it grants degenerate coordination and control solutions for the motor problem).
(c) Motor creativity is granted by adaptive movement variability, at both the levels of coordination and control.

**FIGURE 2:**
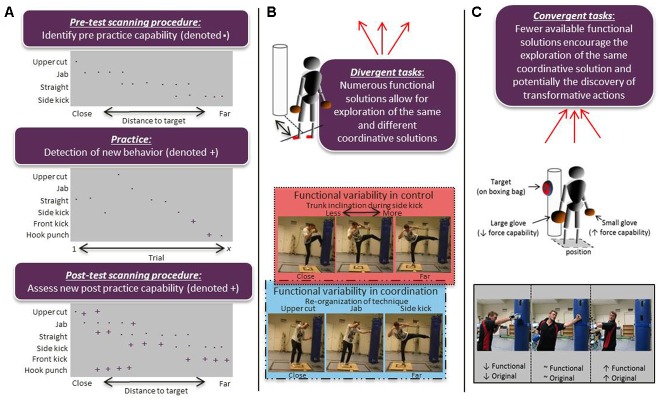
Understanding and assessing motor creativity at the coordination and control levels. Examples for the use of scanning procedures in experimental design for identifying new behaviors **(A)**, designing divergent tasks **(B)**, and convergent tasks **(C)**.

To investigate motor creativity, methodological strategies must evolve around using motor tasks that invite participants to actively search solutions to a motor problem across a series of attempts, such as during practice. In each case, a scanning procedure is necessary to gauge movement variability. Specifically, in order to assess what participants are capable of prior to and after practice, dynamical systems frameworks propose the use of a scanning procedure (Kostrubiec et al., 2012). In scanning procedures, the participant is, under the systematic manipulation of constraints, exposed to a perceptual-motor workspace in order to observe the influence on the formation of coordination and control solutions (Smith and Thelen, 2003). Prior to practice the amount of stable coordination and control solutions uncovered using a scanning procedure gives an estimation of the movement variability in a participant’s behavioral repertoire (Zanone and Kelso, 1992). During practice, a participant might discover and exhibit new actions relative to the scanning procedure pre-test. Furthermore, after practice, whether the individual has acquired new coordination and control solutions can be assessed, again using a scanning procedure (exemplified in **Figure 2A**). Identifying new solutions that meet a criterion for task success (functionality) and have statistical level of rarity for the particular workspace (originality) is a straightforward and theoretically consistent methodology for studying motor creativity. For instance, machine learning methods, such as cluster-analysis, are one way to classify distinct coordination and/or control solutions based on the degree of similarity and dissimilarity amongst a distribution of movement patterns (Rein et al., 2010), after which their statistical rareness can be assessed within and between participants (Memmert and Perl, 2009).

In cases where group or practice interventions are compared, scanning procedures may also be conceptualized as analogous to divergent tasks. To exemplify, **Figure 2B** shows how by systematically varying the distance between a participant and a boxing bag, the variability in coordination solutions (for example hooks, jabs or kicks) that the participant can adopt can be observed and how the effectiveness of each action can be assessed, such as in terms of impact force. Furthermore, motor tasks also provide research opportunities to design problems with the possibility for fewer effective solutions, potentially analogous to convergent and insight tasks (see, Kounios and Beeman, 2014). For example, recalling in **Figure 1**, a key constraint under which the ‘Fosbury flop’ emerged in high jump was the introduction of foam safety mats, allowing safe exploration and practice of landing on the back. Understanding how such an adaptation emerged is a pertinent goal for creativity research. Motor tasks allow observing the exploration to satisfy (changing) constraints during which new creative actions emerge. **Figure 2C** exemplifies how studying transformative motor solutions, such as a qualitative change in technique that leads to improved performance, might be operationalized. The figure depicts how, by requiring an individual to strike a target placed high and on the right side of a boxing bag, and, by placing extra padding on the individual’s right hand, may or may not invite a spinning left back hand as a highly novel and functional way to strike the target. Of course other motor tasks contain ample opportunity to study creative action along similar lines.

### Future Questions

We have considered how changing constraints induces exploration and fosters adaptive movement variability at the levels of coordination and control, particularly in deliberate practice. Indeed, the enhanced movement variability grants creative motor actions. However, many (empirical) questions remain. Following Simonton (2003), we define creative motor action largely as a statistical property of movement variability, and argued against an inherent creative cognitive system or pathway. Instead, we claimed that creative coordination and control solution emerge *in* action. This points to the structure of exploration to satisfy (changing) constraints as being crucial in discovering new movement solutions.

Outstanding questions are, does an increase in an individual’s exploratory actions actually lead to a greater statistical likelihood for increased creative motor actions? Does the nature of this exploration (i.e., the way the workspace is searched) affect the likelihood for increased creative motor actions, also in terms of new coordination and control solutions? Are individuals more likely to find creative coordination solutions when they are not deliberately searching new solutions? How do person constraints (e.g., working memory capacity, motor skill) and task and environmental constraints (e.g., divergent versus convergent tasks, practice interventions such as type and amount of instruction and feedback) affect the structure of exploration (Moraru et al., 2016)?

For example, Nonaka and Bril (2012, 2014) analyzed the hammering behavior of craftsmen making stone beads. With a hammer, the craftsmen strike a raw stone three to four times each second, while they simultaneously reposition the stone with the other hand. The strikes vary from one repetition to the other in amplitude and pace. Using fractal-analysis, the authors show that the variability in the oscillating wielding movements is not random, but correlated over time scales of different length. Presumably, similar fractal-analysis can also be used to describe the nature of exploration during practice, and measure, for instance, the degree to which the variability in different practice attempts correlate, and how this affects the likelihood for finding new coordination and control solutions. In the end, this may provide crucial insight whether this process can be reliably promoted in individuals during practice.

## Concluding Remarks

We have challenged the common assumptions that creativity reflects an internal process of generating ideas underpinned by distinct cognitive creativity systems. In our view, creative solutions emerge *in* action. To satisfy (changing) constraints, the individual is invited to explore the workspace, resulting in increased adaptive movement variability. This increased movement variability is at the heart of understanding the emergence of creative motor actions both at the level of coordination and control. Hence, rather than invoking separate cognitive systems for ideation, we have set out an operational approach to test how constraints coalesce to induce movement variability. We have argued that creative motor action reflects new, in the sense of statistically rare, and adaptive coordination and/or control solutions. The individual’s movement (re)organization following a systematic manipulation of constraints provides a vehicle toward testing how and why creative motor actions emerge. In particular, we suggest future research should focus on the questions surrounding how to structure exploration and induce variability within the learning context to enhance creativity.

## Author Contributions

DO, JvdK, DM, and GS: contributed to conceptualization, background research, and draft work.

## Conflict of Interest Statement

The authors declare that the research was conducted in the absence of any commercial or financial relationships that could be construed as a potential conflict of interest.
